# Growth and function of equine endothelial colony forming cells labeled with semiconductor quantum dots

**DOI:** 10.1186/s12917-018-1572-3

**Published:** 2018-08-23

**Authors:** Randolph L. Winter, Wen J. Seeto, Yuan Tian, Fred J. Caldwell, Elizabeth A. Lipke, Anne A. Wooldridge

**Affiliations:** 1 Department of Clinical Sciences, Auburn University, College of Veterinary Medicine, Auburn, AL USA; 2 Department of Chemical Engineering, Auburn University, Auburn, AL USA

**Keywords:** Qtracker, Regenerative medicine, Horse, Endothelial progenitor cells

## Abstract

**Background:**

Endothelial progenitor cells (EPCs) contribute to neovascularization and vascular repair in vivo and are attractive for clinical use in ischemic disease. Tracking of stem and progenitor cells is essential to determine engraftment after administration. Semiconductor quantum dots (QD) are promising for cell labeling due to their ease of uptake by many cell lines and their continued presence after many cell generations. The purpose of this study was to evaluate function and growth of equine EPCs after QD labeling. Additionally, this study evaluated the duration of QD label retention and mechanisms of QD label loss.

**Results:**

Endothelial colony forming cells (ECFCs) from adult horses (*N* = 3) were employed for this study, with QD labeled and unlabeled ECFCs tested from each horse. Cell proliferation of ECFCs labeled with QD at 20 nM was quantified by comparing the number of cell doublings per day (NCD) and the population doubling time (PDT) in labeled and unlabeled cells. Function of labeled and unlabeled ECFCs was assessed by comparing uptake of acetylated low-density lipoprotein (DiO-Ac-LDL) and tubule formation on growth factor containing matrix. Cell proliferation was not impacted by QD labeling; both NCD (*p =* 0. 95) and PDT (*P =* 0. 91) did not differ between unlabeled and QD labeled cells. Function of ECFCs assessed by DiO-Ac-LDL and tubule formation was also not different between unlabeled and QD labeled cells (*P =* 0. 33 and *P =* 0. 52, respectively). ECFCs retained their QD labeling over 7 passages with both 5 nM and 20 nM label concentrations. Reduction in label intensity was observed over time, and the mechanism was determined to be cell division.

**Conclusions:**

Equine ECFCs are effectively labeled with QD, and QD concentrations up to 20 nM do not affect cell growth or function. QD label loss is a result of cell division. The use of QD labeling with equine EPCs may be an ideal way to track engraftment of EPCs for in vivo applications.

## Background

Quantum dot (QD) nanocrystals are fluorescent cell labeling particles composed of semiconductor nanocrystals typically consisting of a heavy metal core such as CdSe coated with a high band-gap semiconductor such as ZnS [1]. QD nanocrystals are internalized into the cytoplasm of cells with an enzyme-independent mechanism; therefore, they are used to label a variety of cell types in vitro [2]. QD nanocrystals have a high fluorescent efficiency and minimal tendency to photobleach making them desirable for cell labeling in in vitro and in vivo applications [1, 2]. The use of QD labeling also has the advantage of multiple color spectra for labeling different cell types and longevity of the label for in vivo cell tracking. Both short-term tracking of QD-labeled cells over 7–10 days, as well as longer term tracking of QD-labeled cells up to 24 weeks have been demonstrated in multiple different cell types [3–6]. In addition to the imaging advantages, QD labeled cells do not transfer their QD label to surrounding cells with cell death [1, 2, 7, 8]. Although the mechanism of QD label loss differs between cell types and must be tested for each new cell type, typically cells do not metabolize the QD label, meaning that labeled cells retain the QD and also pass the QD on to subsequent progeny cells during cytoplasmic partition with cell division [2, 7].

Tracking of stem and progenitor cells is essential to determine engraftment after administration. Multiple types of cell labeling strategies have been employed for in vitro and in vivo tracking including cell transduction with subsequent green fluorescent protein expression, superparamagnetic iron oxide (SPIO) nanoparticles, and QD nanocrystals [9–11]. Important criteria when assessing the potential of a cell labeling strategy for use include ease of use, efficacy of label, and ability to maintain cellular phenotype after label. Outcomes frequently differ between cell types and between species. Therefore, detailed in vitro investigation is critical prior to use for in in vivo cell tracking experiments. Concerns exist over the possibility of replication-competent lentivirus with transduction, and identification of SPIO containing cells ex vivo using Prussian blue stain has specificity problems [9, 10]. In recent years, QD labeling has gained prevalence for use and has been used in many different cell types (including human endothelial progenitor cells) and species [2–4, 7]. Different mechanisms of label loss exist among the cell types studied [2, 7]. In the mouse, one study showed that embryonic fibroblasts lost QD label from cell proliferation, but embryonic stem cells lost QD label at least in part from label degradation and excretion [2]. In another study of mouse embryonic stem cells labeled with QD, a loss of QD label was suggested to occur from either cell division or leaking of the label from the cell [9, 12]. A study using human endothelial progenitor cells suggested that QD label loss occurred primarily from cell division [7].

The recent isolation and in vitro work with equine endothelial progenitor cells (EPCs) and their subset endothelial colony forming cells (ECFCs) is promising for the in vivo, regenerative use of ECFCs as therapeutic agents [10, 11, 13, 14]. Although QD labeling has been used for cell tracking of equine mesenchymal stem cells [15, 16], there are no studies evaluating the effects of this label on function of equine ECFCs or mechanism of label loss. Prior to in vivo vascularization studies using these QD-labeled ECFCs, it is important to know how ECFCs function after QD label, the duration of ECFC label retention and how these cells lose their label. Thus, the objectives of this study were to evaluate cell growth and cell function in equine ECFCs in vitro after QD-label. Additionally, the mechanism of QD label loss in equine ECFCs was investigated.

## Methods

### Isolation, storage, and classification of ECFCs

All protocols involving animals were approved by the Auburn University Animal Care and Use Committee, protocol # 2014–2408. Whole blood was collected from three, healthy, university-owned adult horses aged 15–26 from either the cephalic vein or the jugular vein for ECFC isolation using either a whole blood isolation or density gradient centrifugation isolation method as previously described [13, 14]. The ECFCs were cryopreserved at a concentration of 100,000 cells / mL in a freezing medium containing 95% equine serum and 5% dimethyl sulfoxide and then stored in liquid nitrogen as passage 2 cells. ECFCs were thawed and used for all experiments at passage 3–5. ECFCs were cultured in either 25 cm^2^ or 75 cm^2^ tissue culture polystyrene flasks in endothelial cell growth medium with manufacturer-supplied growth factors and anti-microbials (EGM-2 with Bullet Kit, Lonza, Visp, Switzerland) with equine serum at a final concentration of 10% (HyClone Laboratories Inc., Logan, UT, USA) at standard cell culture conditions (37 °C, 5% CO_2_, 95% humidity). ECFCs were defined as late-outgrowth EPCs based on characteristic cobblestone morphology and rapid in vitro cell division [13]. The ECFCs used in this study expressed CD34, CD105, VEGFR-2, vWF, and CD14, formed vascular tubules on basement membrane and also had receptor-mediated uptake of fluorescently-labeled acetylated low-density lipoprotein (DiO-Ac-LDL) (Biomedical Technologies Inc., Stoughton, MA, USA), consistent with our group’s previous work [13].

### Cell labeling

ECFCs were labeled with fluorescent QD with an emission maxima at 655 nm (Qtracker® 655 Cell Labeling Kit, Invitrogen, CA, USA) based on manufacturer instructions for labeling adherent cells. Briefly, the QD nanocrystals were added to the manufacturer’s supplied carrier of phosphate buffered saline (PBS) and incubated at room temperature for 5 min. Fresh complete growth medium was then added, and then vortexed for 30 s. Cell culture medium was removed from adherent cells in a 25 cm^2^ cell culture flask, the cells were washed with PBS, and then the QD containing mixture was added to the cell culture flask.

### Measurement of cell growth after QD label

For cell growth experiments, ECFC lines from 3 horses (*N* = 3) were tested individually. ECFCs from passage (P)3 were seeded into collagen-coated (50 μg/mL) 25 cm^2^ cell culture flasks with 75,000 cells (3000 cells/cm^2^) for each QD-labeled and control condition. Once the cells were ~ 50% confluent, they were labeled with 20 nM QD or left unlabeled for 24 h. After reaching 80% confluency, cells were subcultured by adding trypsin-EDTA at 0.25 mg/mL (Lonza, Visp, Switzerland) and incubating at 37 °C for 1 min. Trypsin was neutralized with an equal volume of fresh ECFC culture medium, followed by centrifugation at 200 x g for 5 min. Cells were then reseeded onto collagen-coated 75 cm^2^ cell culture flasks with 225,000 cells per flask (3000 cells/cm^2^). Cell seeding density after each subculture, cell number at the time of subculture, and time (hours) between subcultures were recorded and used to determine the number of cell doublings (NCD) in each 24 h period as well as the population doubling time (PDT) in hours. Additionally, the cumulative population doubling level (CPDL) was determined from the cell count. Cell counts were performed using standard cell culture protocol where 4 fields were counted on the hemocytometer and then averaged before applying the dilution multiplier to obtain the final cell counts. This was performed in triplicate at all subcultures. NCD was calculated as: $\mathrm{NCD}=\left[\frac{\log 2\frac{\mathrm{CH}}{\mathrm{CS}}}{\mathrm{Number}\kern0.17em \mathrm{of}\kern0.17em \mathrm{days}}\right]$, where C_H_ was the number of cells at the time of subculture and C_S_ was the number of cells seeded. PDT was calculated as: $\mathrm{PDT}=\frac{\mathrm{Total}\kern0.17em \mathrm{number}\kern0.17em \mathrm{of}\kern0.17em \mathrm{hours}}{\mathrm{NCD}}$. The CPDL was calculated as: $\mathrm{PDL}=\frac{\log 10\left(\mathrm{CH}\right)-\log 10\left(\mathrm{CS}\right)}{\log 10(2)}$. The calculated PDL for each subculture was then added to the previous subculture PDL to determine CPDL. Cells were subcultured at 80% confluency to ensure that the cells remained in log growth. Cells were subcultured until P10.

### Quantification of QD label

To quantify the amount of QD label over multiple cell passages using flow cytometry, each of the ECFC cell lines at P3 from 3 horses were subjected to the same labeling procedures as described above and the following conditions: unlabeled cells, 5 nM, and 20 nM QD. Subculture procedures were performed as described above. After labeled cells reached 80% confluency cells were reseeded onto collagen-coated 25 cm^2^ cell culture flasks at a 1:4 ratio. Cells were subcultured in this manner out to passage 10.

At each passage, cells that were not used to seed the cell culture flasks were washed with 10 mL of calcium-free, magnesium-free PBS solution and then centrifuged at 200 x g for 5 min. The PBS solution was removed, 1 mL of 4% paraformaldehyde solution was added, and the cells were incubated at room temperature (25 °C) for 20 min. Cells were then centrifuged at 200 x g for 5 min, washed with PBS solution, resuspended in 1% Bovine serum albumin (BSA) solution (OmniPur, Gibbstown, NJ, USA), and kept at 4 °C. Immediately prior to flow cytometry analysis, the fixed cells were filtered through a 35-μm mesh. Using a 675–25-H flow cytometry filter, a total of 15,000 events were collected for each sample, with forward scatter versus side scatter plots used for imaging (BD Accuri C6 flow cytometer, BD Biosciences, Brea, CA, USA). Gates were set to select for live cultured cells, with elimination of doubled cells, dead cells, and debris (BD Accuri C6 software, BD Biosciences, Brea, CA, USA).

### Assessment of cell function after QD label

ECFCs from P3 from each horse cell line (*N* = 3) were seeded at 75,000 cells per flask into two 25 cm^2^ cell culture flasks, cultured until 50% confluency, and then left unlabeled or labeled with 20 nM QD. Once cells were ~ 80% confluent, they were subcultured using trypsin-EDTA as described above and used in either tubule formation assays or DiO-Ac-LDL uptake assays. A 96-well cell culture disk was prepared with 75 μL/well of solubilized basement membrane (BD Matrigel Basement Membrane Matrix, BD Biosciences, Bedford, MA, USA), which was incubated for 30 min at 37 °C prior to cell seeding. Each well was seeded with 10,000 cells and then incubated at 37 °C. Vascular tubule formation was assessed at 24 h post seeding. Three replicates of duplicate assays were performed for each horse cell line. The presence or absence of tubule formation was noted using light microscopy. Tubule quality score was subjectively scored as previously described [13] (1 = no tubule formation, 2 = tubules projecting from cells but no connections between cells, 3 = vascular tubule formation with connecting tubules in ≤ 50% of the field, and 4 = vascular tubule formation with connecting tubules in > 50% of the field). Tubule quality scores were assessed by one investigator (RLW) who was blinded to cell labeling conditions.

ECFCs labeled with 20 nm QD and unlabeled ECFCs were seeded in duplicate at a density of 50,000 cells/well into 12-well cell culture plates that had been previously coated with collagen. Once cells were ~ 50–80% confluent, DiO-Ac-LDL, diluted in prewarmed supplemented medium, was added to a final concentration of 50 μg/mL. Cells were incubated with DiO-Ac-LDL for 4 h at standard cell culture conditions. After incubation, the cells were washed three times with fresh cell culture medium. Cells were then harvested with a Trypsin-EDTA solution and centrifuged at 200 x g for 5 min. Cells were washed with PBS solution and fixed with 4% paraformaldehyde as described above. Cells were then washed with PBS, resuspended in 1% BSA solution, and filtered through a 35-μm mesh immediately prior to flow cytometric analysis. DiO-Ac-LDL has an emission peak at 507 nm, so a FLH-1 flow cytometry gate was used for analysis. A total of 15,000 events were collected for each duplicate sample, with forward scatter versus side scatter plots used for imaging. Gates were set to select for live cultured cells, with elimination of doubled cells, dead cells, and debris.

### Determining mechanism of QD label loss

ECFCs from each horse cell line (*N* = 3) were seeded as P4 in a 25 cm^2^ cell culture flask and labeled with 20 nM of QD or left unlabeled as described above. Once these cells were ~ 80% confluent, they were subcultured at 150,000 cells/well (40,000 cells/cm^2^) into 12-well cell culture plates previously coated with collagen. Four wells were seeded for each test condition which included: unlabeled cells with and without a growth inhibitor and 20 nM QD labeled cells with and without a growth inhibitor, mitomycin C (MMC) (Sigma-Aldrich, MO, USA). At initial seeding of the wells, the growth inhibited wells contained supplemented cell culture medium with 20 μg/ml MMC. After 24 h in standard cell culture conditions, the cell culture media was removed and replaced with either standard cell culture medium or MMC-containing supplemented cell culture medium as appropriate. Cells were harvested from two wells per condition after 24 h using Trypsin-EDTA. These cells were prepared for flow cytometry and analyzed for QD quantification as described above. After an additional 24 h in standard cell culture conditions, the remaining cells were harvested and analyzed with flow cytometry.

### Statistical analysis

All statistical analyses were performed with commercially available statistical software (JMP®, Version 13.0.0 SAS Institute Inc., Cary, NC, USA). Data were assessed for normality using a Shapiro-Wilk test. Cell growth data with a normal distribution were expressed as mean ± SD and compared using either a Student’s t-test or ANOVA. The decline in QD fluorescence data and CPDL with nonparametric distributions were expressed as median [range] and compared using a Wilcoxon rank sums or Kruskal Wallis test. Tubule formation categorical data were analyzed by a Fischer’s exact t-test. Significance was assessed as a *p* value < 0.05.

## Results

### QD effects on cell growth

**Fig. 1:**
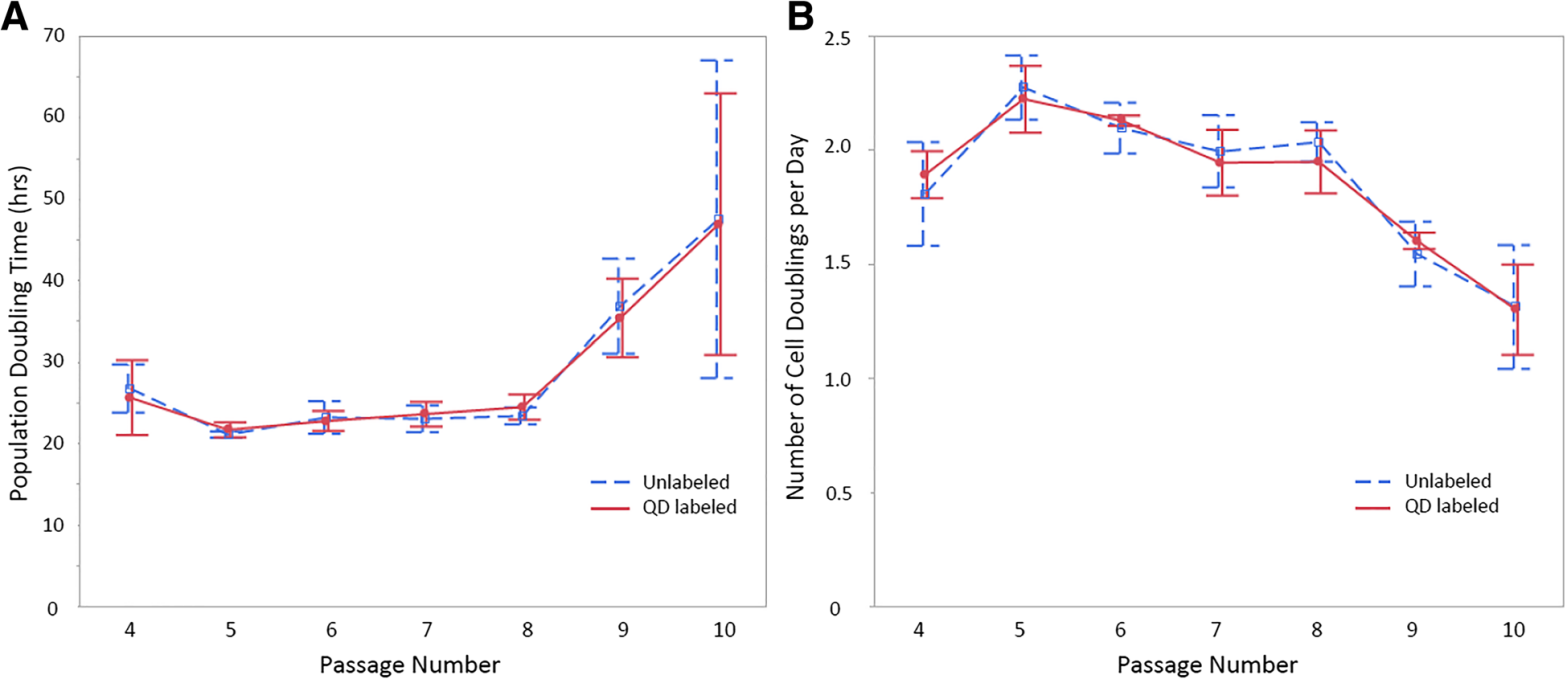
**a** Population doubling time in hours and **b** number of cell doublings per day by passage for unlabeled ECFCs and ECFCs labeled with 20 nM QD. Each time point is the mean ± SD of data from 3 horses

Cell growth parameters were not different between QD labeled and unlabeled cells at any passage. NCD, PDT, and CPDL data were assessed in QD labeled P4 – P10 ECFCs for all horse cell lines (*N* = 3). NCD for unlabeled ECFCs were not significantly different compared to QD-labeled ECFCs (*P =* 0.95), indicating that QD label did not affect the NCD. PDT for unlabeled ECFCs was not significantly different compared to QD-labeled ECFCs (*P =* 0.91), indicating that QD label did not affect the PDT. The maximum CPDL at P10 for unlabeled ECFCs (27.9 [26.14–28.48] cell doublings) was not different compared to QD-labeled ECFCs (28.27 [25.97–28.3] cell doublings, *P* = 0.83). NCD and PDT in both labeled and unlabeled cells by passage number are shown in Fig. 1.

### Quantification of QD over cell passages

**Fig. 2:**
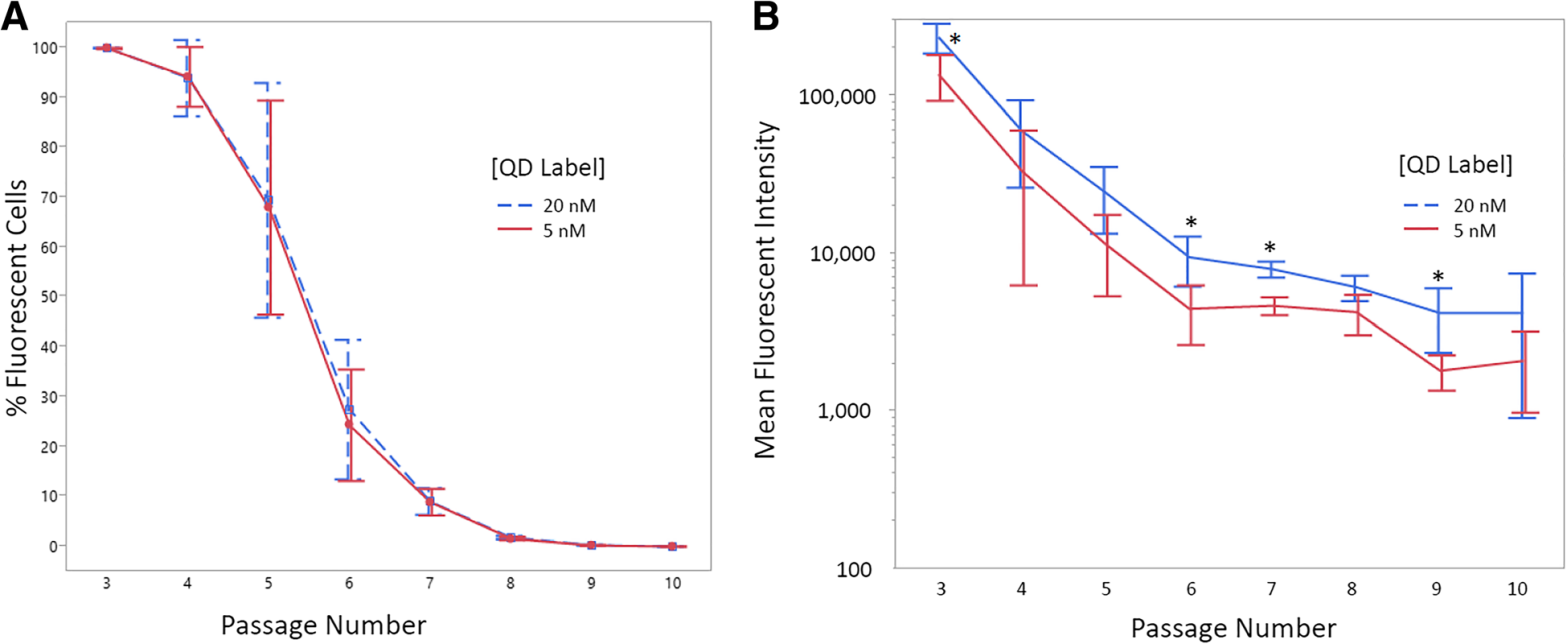
**a** Percentage of cells fluorescent labeled (% fluorescent cells) and **b** Decrease in mean fluorescence intensity by cell passages in ECFCs (*N* = 3) over time for 5 nM and 20 nM QD label concentrations. Data are displayed as mean +/− SD

Flow cytometry was used to determine the percentage of QD labeled ECFCs by passage and the mean fluorescent signal intensity from P3-P10 (Fig. 2). ECFCs labeled with 5 nM had a similar decline in the percentage of labeled cells as ECFCs labeled with 20 nM (Fig. 2) with 100% labeled at P3 and almost 0% labeled at P10. Although there were no differences in the percentage of cells labeled between 5 nM and 20 nM QD, the 20 nM QD labeled ECFCs had a significantly greater mean fluorescent signal at P3 (flow cytometric analysis performed immediately after the 24 h label contact period at the initial labeling), P6, P7, and P9 (*P =* 0.035, *P =* 0.031, *P =* 0.003, *P =* 0.27, respectively) compared to the 5 nM QD labeled ECFCs (Fig. 2).

### Cell function after QD label

**Fig. 3:**
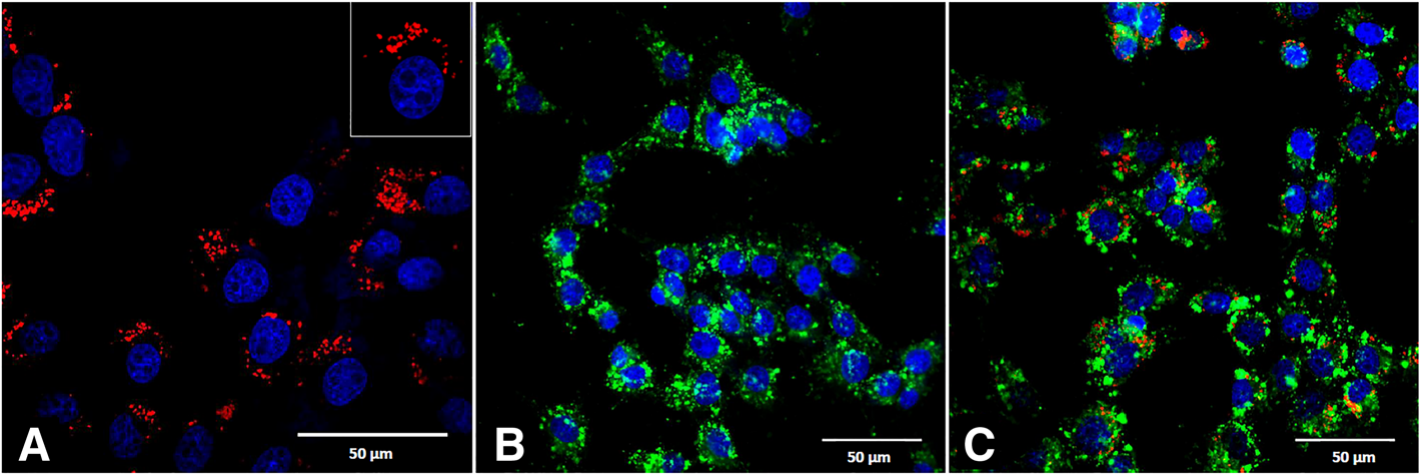
Representative photomicrographs from 3 equine ECFC cell lines (merged images) showing **a**) quantum dot (QD, red) labeled equine ECFCs (an enlarged image of one cell is in the upper right corner); **b**) ECFCs not labeled with QD demonstrating cellular uptake of DiO-Ac-LDL (green) and **c**) QD labeled (red) ECFCs demonstrating cellular uptake of DiO-Ac-LDL (green). Nuclei are stained with DAPI (blue). Note the similar uptake of DiO-Ac-LDL in labeled and unlabeled ECFCs. Scale bars are 50 μm

The ability of ECFCs to uptake LDL and form tubules in vitro was not affected by the QD label. Flow cytometry was used to assess the percentage of unlabeled ECFCs and of 20 nM QD labeled ECFCs that had DiO-Ac-LDL uptake in all horse cell lines (*N* = 3) at P4. The percentage of ECFCs with DiO-Ac-LDL uptake was 99.17% ± 0.45% for unlabeled cells and 98.93% ± 0.68% for QD labeled cells, with no significant differences (*P =* 0. 33). A representative photomicrograph of the uptake of DiO-Ac-LDL by unlabeled ECFCs and QD labeled ECFCs is shown in Fig. 3, and the cytoplasmic localization of QD label is also evident in this figure.

**Fig. 4:**
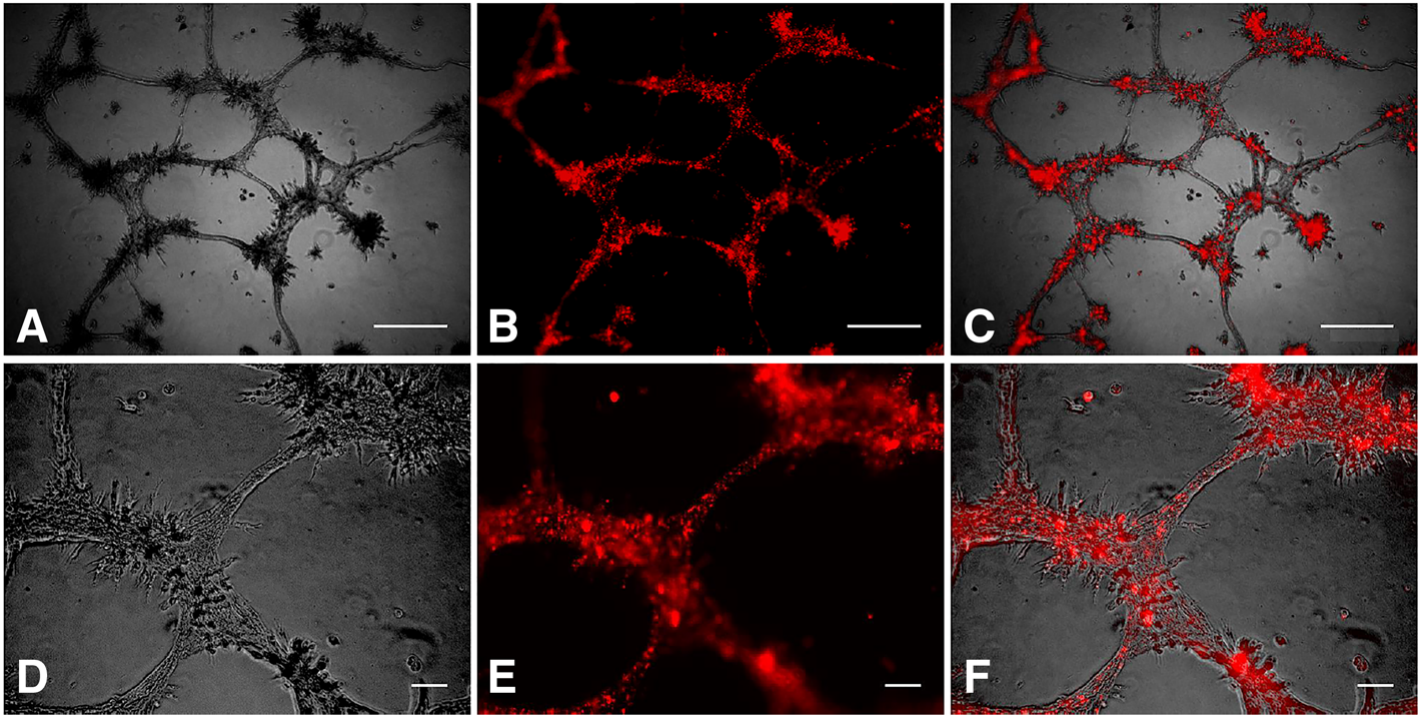
Representative photo micrographs of in vitro tubule formation in QD-labeled ECFCs (red) from 3 horses. Three replicates of duplicate assays were performed for each horse cell line. Panels **a** and **d** are light photo micrographs. Panels **b** and **e** are fluorescent photo micrographs. Panels **c** and **f** are merged images. Scale bars are 500 μm

ECFCs, both unlabeled and QD labeled, were seeded onto basement membrane matrix as described above, and photomicrographs were used to score tubule quality in all horse cell lines (N = 3). Three replicates of duplicate assays were performed for each horse cell line. The range of tubule scores in both groups was 3–4, and there was no significant difference in tubule quality score between unlabeled and QD labeled ECFCs (*P =* 0.524), indicating that the presence of QD label does not inhibit tubule formation (Fig. 4).

### Mechanism of label loss

**Fig. 5:**
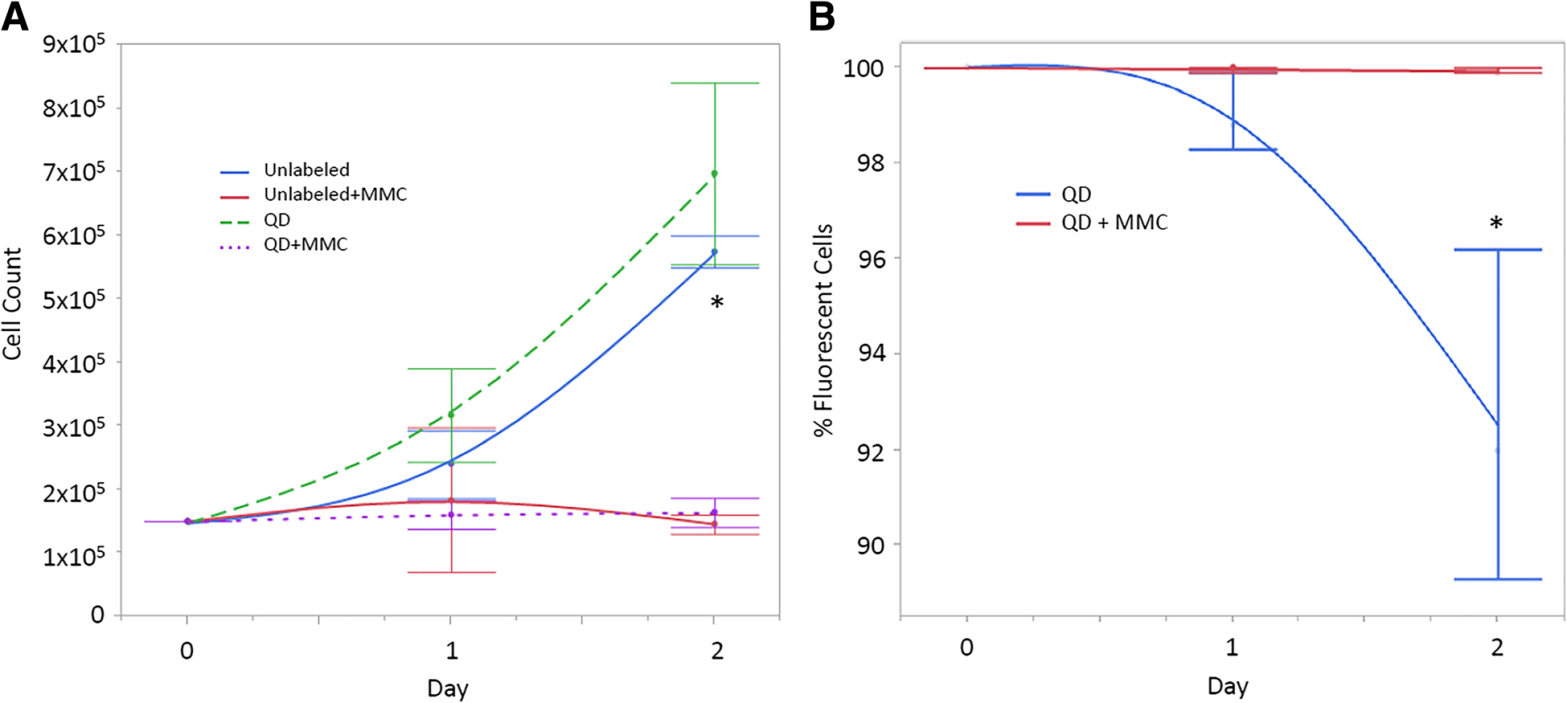
**a**) Cell counts for unlabeled and QD-labeled cells with and without the cell division inhibitor mitomycin C (MMC). *Indicates a significant difference between groups (*p* < 0.05). **b**) Decline in the percentage of QD-labeled cells in groups with and without MMC

**Fig. 6:**
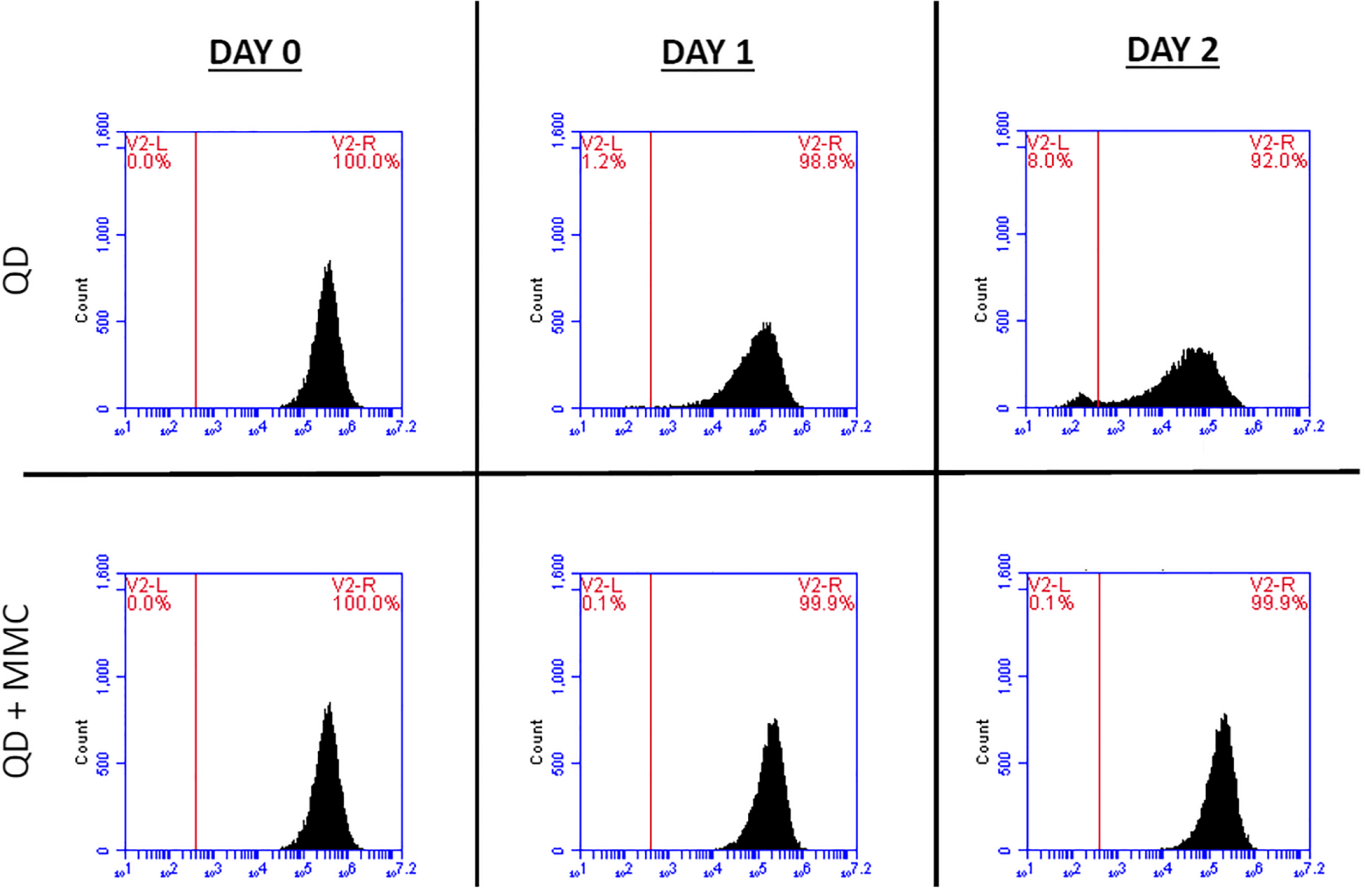
Histograms of flow cytometric analysis of QD-labeled cells with and without the growth inhibitor mitomycin C (MMC). QD-labeled cells without MMC (top row) have a decline in the percentage of labeled cells over two days of cell division compared to cells not undergoing cell division (bottom row)

ECFCs seeded with a growth inhibitor maintained QD label longer than uninhibited cells showing that cell proliferation is the primary cause of QD label loss in ECFCs. Treatment with the cell division inhibitor MMC caused a significantly lower cell count in both unlabeled (*P =* 0.0005) and QD labeled (*P =* < 0. 0001) cells versus untreated cells. There was no difference in cell counts on day 2 between unlabeled ECFCs and QD labeled ECFCs with (*P =* 0.99) or without (*P =* 0.252) MMC. The quantity of QD label was significantly higher (*P* = 0.007) in MMC treated cells versus untreated QD labeled cells (Fig. 5). The flow cytometry data shows the decline in the QD label in cell undergoing cell division versus those that are not (Fig. 6.)

## Discussion

Using labeled cells for cell tracking in vitro or in vivo studies for disease models is essential for cell localization and evaluation [17–20]. The engraftment and differentiation of an injected/implanted stem cell can then be specifically determined rather than just speculated [21, 22].

This in vitro study of equine ECFCs labeled with QD revealed that there are no adverse consequences of the label on cell growth or function. Using QD label on cells intended for injection into an animal should be considered a reliable way to monitor the location and activity of the ECFCs [4–6]. As cellular therapy and regenerative medicine continue to advance, understanding the activity and location of any injected cells is paramount to understanding the true effectiveness of these injected cells [9]. Good study design which includes appropriate control groups will assist in this assessment, but the value of having the ability to objectively identify the location of a previously injected cell, either in vivo or ex vivo, cannot be understated. Based on the strong fluorescent signal emitted at many time points subsequent to the labeling process, using a QD label in equine ECFCs should be considered a reasonable option to investigators.

In investigating regenerative cellular therapy, QD labels are used extensively in animal models for human and veterinary medicine [4, 6, 12, 23]. Other methods of cell labeling have been used in equine disease models such as transduction with lentivirus to induce green fluorescent protein production or in vitro labeling with SPIO [9–11]. QD labels are easy to apply, have been shown to be safe in multiple cell lines, and are readily identifiable in cells long after the label is applied. Some studies suggest that the outer coating used in QD formation as well as the concentration used may affect how toxic QD is to cells [11, 24]. Lower concentrations of QD label are generally thought to be less toxic, which is why some studies use relatively lower concentrations of QD label [7, 11]. The coating for the QD nanocrystals used in this study is a CdSe coating, similar to many other commercially-available QD labels. We assessed cell function immediately after the 24 h incubation with the QD label in equine ECFCs, which would be the time point at which the QD label concentration was greatest. No adverse effects on cell function, cell growth, or cell morphology up to P10 were observed in QD labeled cells in this study. Therefore, using a QD label concentration of up to 20 nM in equine ECFCs would allow these cells to be tracked without adversely affecting cell behavior. The concentrations of QD used in this study were chosen based on preliminary work in the authors’ lab.The QD concentrations of 5 nM and 20 nM were chosen based on the ability to subjectively differentiate the fluorescence intensity of these concentrations, which was considered useful for in vivo applications of tracking cells in tissues, and using concentrations over 20 nM was unlikely for in vivo applications. However, comparison of proliferative capacity and cell function at multiple other doses was not performed in the current study.

Using a QD label of 8 nM on human ECFCs at standard cell culture seeding and cell division conditions, less than 20% of the QD label remained after 3 passages [7]. In our study using a 20 nM QD label concentration on equine ECFCs, we observed 27.5% +/− 14% label remained after 3 passages. These cultured cells have an optimal environment for cell division. Quantification of the differences between in vitro and in vivo cell growth conditions is not straightforward, and there are many microenvironments that may not be optimized for ECFC division. For instance, cells that are in vivo experience reduced nutrients and space compared to in vitro standard cell culture environments [25–28]. This may be particularly true for pro-inflammatory in vivo environments where tissue oxygen and metabolite levels are decreased [26–28] . Cell function has been shown to be compromised for EPCs and other cells within inflammatory conditions [26, 27] . Therefore, ECFCs would divide less over a given amount of time compared to that same time in vitro cell culture conditions.

Some studies using a QD label report a variable labeling efficiency in different cell lines, but this study shows that equine ECFCs from multiple lines are all labeled efficiently with QD. For studies investigating autologous cells for regenerative therapies, consistency of QD labeling between lines is critical to obtaining meaningful results. The mechanism of label loss varies by cell type. One study demonstrated that QD label was rapidly degraded by murine embryonic stem cells, with 15. 8 ± 2. 9% of embryonic stem cells maintaining QD label only 48 h after label [2]. This is in contrast with murine embryonic fibroblasts which maintained high levels of QD labeled cells 48 h after label, with almost 95% of embryonic fibroblasts maintaining QD label after 72 h when MMC was added to cell culture media [2]. Mouse embryonic stem cells were also found to excrete QD label into the surrounding cell culture media [2]. Human endothelial progenitor cells may lose QD label from cell division, and mouse embryonic stem cells lose QD label by cell division or leaking of the QD label [7, 12]. In our study, we investigated the mechanism of label loss by blocking cell division through the addition of MMC. If QD label leakage or metabolism was the major mechanism of label loss in equine ECFCs, then the percentage of cells labeled with QD or the fluorescent intensity would have decreased over time in cells with and without MMC added. However, a substantial label loss was not observed when MMC was present. Therefore, our study shows that equine ECFCs have substantial loss of QD label over time due to cell division.

## Conclusion

This study demonstrates that equine ECFCs are labeled effectively with QD nanocrystals, but label loss occurs as the label is partitioned to daughter cells during division. Equine ECFCs do not have their growth or functional characteristics altered with QD label, thus making this in vitro labeling procedure favorable for in vivo cell tracking of this cell type in horses.

## Abbreviations

BSA: Bovine serum albumin
C_H_: Number of cells at time of subculture
C_S_: Number of cells seeded
DiO-Ac-LDL: 3,3′-Dioctadecyloxacarbocyanine-acetylated low density lipoprotein
ECFCs: Endothelial colony forming cells
EPCs: Endothelial progenitor cells
MMC: Mitomycin C
NCD: Number of cell doublings per day
P: Passage
PBS: Phosphate buffered saline
PDT: Population doubling time
QD: Quantum dots
SPIO: Superparamagnetic iron oxide particles
